# Supplementary material for: Are carbon emissions trading and green financial instruments synergistic? -Comprehensive quantitative research based on content analysis
Source: PLoS One. 2024 Mar 7;19(3):e0298601. doi: 10.1371/journal.pone.0298601 (PMC10919601; doi:10.1371/journal.pone.0298601)
Supplement: S1 Data — (ZIP) [file pone.0298601.s001.zip › Data/地级市Z_F工作报告.zip!/╣π╢1⁄2/╒╪╟∞╩╨/└┤╫╘╣1⁄2╓┌║┼í╛┬φ┐╦╩2╛▌═°í┐_2014.docx]

各位代表：

　　我代表肇庆市人民政府向大会作工作报告，请予审议，并请政协委员和其他列席人员提出意见。

　　2013年工作回顾

　　去年，面对严峻复杂的经济形势，在省委、省政府和市委的正确领导下，在市人大及其常委会和市政协的监督支持下，市政府全面贯彻党的十八大精神，紧紧围绕把肇庆建设成为珠三角连接大西南枢纽门户城市的奋斗目标，全面落实《珠三角规划纲要》，大力实施“两区引领两化”战略，全力抓发展、促转型、增后劲、惠民生、保稳定，圆满完成市十二届人大三次会议确定的目标任务，呈现出新的发展亮点：固定资产投资和金融机构本外币各项贷款余额均超1000亿元，规模以上工业总产值超过3000亿元，被评为全国科技进步考核先进市，粤桂合作特别试验区和广佛肇（怀集）经济合作区发展总体规划通过省政府审批，星湖湿地公园创建成为全省首个国家湿地公园，蝉联全国综治“长安杯”，荣获第十五届省运会承办权。

　　一年来，我们突出抓好发展第一要务，加快推进转型升级，经济发展目标任务全面完成。

　　经济增长稳步提升。出台支持重点骨干企业、中小微企业和外贸企业的政策措施，加大力度推进“六个一批”项目和省市重点项目，经济增长拉动力持续增强，主要经济指标增速居珠三角前列，全年实现地区生产总值1660.07亿元，比上年增长（以下简称增长）11.5%，人均生产总值41479元，增长10.5%；一、二、三产业增加值分别增长5.6%、15.7%、8.5%；完成地方公共财政预算收入120.75亿元，增长16.3%；完成固定资产投资1007.78亿元、外贸进出口总额70.2亿美元、社会消费品零售总额493.12亿元，分别增长20%、10.5%、13.8%；主要旅游景区（点）接待游客2751.43万人次，旅游收入205.87亿元，增长14.7%；商品房销售额231.59亿元，增长31.7%。

　　产业集聚转型加快。出台支持工业主导产业集聚转型政策措施，有82家规模以下工业企业转为规模以上企业，年主营业务收入超10亿元的企业增加到35家，新创建省名牌产品22个，九大主导产业总产值占比提高到67%，实现规模以上工业增加值813.45亿元，增长18.1%，增速居珠三角首位。高新技术企业增加到108家，新组建企业工程中心和技术中心26家，成为国家产学研合作创新示范基地。服务贸易进出口增长91.6%，委托设计和自主品牌混合生产方式出口占比提高到60.5%，外贸出口48.3亿美元，增长27.6%，增速居珠三角首位。积极培育新经济、新业态，端州区成为国家智慧城市试点，华南智慧城被确定为省服务外包示范园区和省物联网重点项目。金融机构本外币各项贷款余额增速居珠三角首位，小额贷款公司增至12家，融资超市为555家企业提供贷款54.37亿元。制定现代农业发展五年行动计划，建成53.8万亩高标准农田，56万亩土地实现流转经营，新发展农业龙头企业42家，新增农民专业合作社数量居全省第一。加快淘汰落后产能，新认定清洁生产企业54家，预计单位生产总值和工业增加值能耗分别下降3%和5%以上。

　　平台建设取得突破。肇庆高新区加快转型发展，引进三浦重工总部等83个优质项目和9个科技孵化项目，创新实施“零土地”招商盘活1710亩闲置土地，与县（市、区）开展“一区多园”合作，连续五年被评为省优秀园区并获评首批省清洁生产示范园区。粤桂合作特别试验区起步区建设全面启动。广佛肇（怀集）经济合作区主干路网基本成形。肇庆（高要）汽车零部件产业园成为省汽车零部件专业园区，成功承办省汽车零部件产业招商推介会。“肇庆金秋”重大发展平台投资洽谈会签约项目63宗，总投资1791.6亿元。全市承接产业转移项目88宗，计划投资188.85亿元。新批外商投资项目129宗，合同外资额31.1亿美元；实际吸收外资12.4亿美元，增长7.8%。

　　重点项目有效推进。100项市重点项目完成投资330.93亿元，完成率为110.7%，其中38项省重点项目投资完成率排名全省第一。广佛肇高速肇庆段全线动工，二广高速怀集至岗坪支线建成通车，贵广、南广铁路肇庆段进入铺轨阶段，广佛肇城际轨道肇庆段完成大部分路基建设，肇庆火车站综合体、鼎湖大道等重点工程启动实施。59项现代产业项目完成投资147.89亿元，星湖新材料铝板带等重点产业项目建成投产。国电热电联产项目试运营，“西电东送”直流输电工程肇庆段建成，省天然气管道肇庆段即将完工。景丰联围加固三四期和封开西江干堤达标加固等水利工程启动建设，完成18宗病险水库除险加固工程。

　　一年来，我们突出抓好城市扩容提质，着力优化宜居环境，城乡建设迈出新的步伐。

　　城市建设有新成效。市主体功能区规划出台实施。完成肇庆市城市总体规划评估报告，编制实施市城区11项专项规划、12项城市设计和控制性详细规划。肇庆新区成为省重大创新平台，产业发展、基础设施、低碳发展专项规划通过省审批，中央绿轴生态城被住建部确定为“国家绿色生态示范城区”，总投资200亿元的11个重点项目进展顺利，引进国际商贸城等优质项目。中心城区建设加快推进，阅江大桥、城东新区城市综合体、广佛肇城际轨道鼎湖站综合开发项目、下湾城市公园等项目顺利实施。启动端州城区城市管理综合行政执法改革，率先在全省创建城市管理“执法通”防控平台。各县（市）新一轮城市总体规划修编全面实施，新城建设和旧城改造同步推进。全市新动工及续建“三旧”改造项目50个，新增动工面积2580.9亩。

　　镇村建设有新面貌。实施创建幸福村居五年行动计划，乡镇总体规划实现全覆盖，村庄规划覆盖率提高到72%。新创建省卫生镇7个、省卫生村215条，建成市级宜居城镇10个、宜居村庄79条、生态文明村100条、宜居社区19个，累计建成名村示范村129条。完成农村公路硬底化402.56公里。建成农村垃圾集中收运“一镇一站”、“一村一点”。

　　生态建设有新进展。制定实施改善环境空气质量综合治理方案，强化建筑陶瓷等行业整治改造。对192家重点污染源企业实行环境信用管理，完成217个污染减排项目和74个重金属污染源综合整治项目。划定乡镇饮用水源保护区75个。及时有效应对贺江水污染事件。完成星湖水质整治外坑渠清淤工程。造林30.5万亩，生态景观林带工程和森林碳汇工程分别完成14.8万亩、8.5万亩，总体进度居全省前列。新建城市绿道慢行道118.7公里。单位建设用地二、三产业增加值每平方公里增加到1.38亿元，被评为省部共建节约集约用地试点示范省先进单位。

　　一年来，我们突出抓好保障改善民生，大力推进基本公共服务均等化，发展成果更多惠及人民群众。

　　人民生活持续改善。公共财政用于11类民生支出132.1亿元，增长10.8%，占预算支出67.6%。投入惠民实事各类资金合计44.25亿元，市十件惠民实事全面完成。城镇新增就业47196人，实现创业3459人，农村劳动力转移就业43162人，城镇登记失业率为2.39%。企业最低工资标准从四类提高到三类。新开工保障性住房5468套，建成3476套，均超额完成任务。发放临时价格补贴5322.89万元，新建平价商店53家、标准化惠民农贸市场12个。居民消费价格指数控制在年度目标以内。预计城镇居民人均可支配收入23929元，农村居民人均纯收入11662元，分别增长10%、12.5%。

　　社会保障更加健全。各项社会保险参保任务全面完成。企业退休人员月人均基本养老金提高到1504元。发行社会保障卡310万张。人力资源和社会保障服务所实现乡镇（街道）全覆盖。城镇职工和城乡居民基本医疗保险年度最高支付额度分别提高到55万元、25万元，实施城乡居民基本医保大病保险，与广州等地医院实现医保异地联网即时结算，医保经办服务网点覆盖所有乡镇（街道）。低保范围扩大到年人均收入低于3000元的农村居民和低于4560元的城镇居民，提高了“五保”和孤儿供养标准。累计投入救灾款物7200多万元。在全省率先完成656户因灾房屋重建。新一轮扶贫开发累计投入帮扶资金2.52亿元。

　　社会事业全面发展。省教育强县（市、区）实现全覆盖，教育强镇覆盖率达92.3%。市第一中学新校区建成使用并创建成为国家级示范性普通高中，义务教育规范化学校覆盖率和普通高中优质学校比例分别达到95%、83.9%。共有4.89万名学生享受中职免学费政策，实施了山区和农村边远地区义务教育学校教师岗位津贴制度。高等教育发展水平不断提高。四会市县级公立医院综合改革试点初见成效。全市各类二级以上医院开展平价医疗服务，基层医疗卫生机构综合改革加快推进，村卫生站全面使用基本药物。建成覆盖市、县、镇的食品安全风险监测网络。解决了16万多人听广播看电视难问题。通过“中国砚都”复评，端砚文化产业园、四会玉器文化产业创意园等项目进展顺利。通过创建全国文明城市年度考核验收。第十二届全运会金牌数居全省第六，成功承办省第三届体育大会并取得全省第四的历史最好成绩。完成省下达的年度人口计划任务。制定了社会组织承接政府职能转移和购买服务目录，建成社会组织孵化基地，登记在册社会组织增至1308家。排查调处各类矛盾纠纷8258宗，成功率达98%。打击各类违法犯罪力度加大，社会治安综合治理得到加强。安全生产形势总体稳定。统计、民族、宗教、外事、侨务、港澳、人防、国防动员、地方志、档案、气象、水文、地震、慈善、妇女儿童、残疾人等工作取得新成绩。驻肇部队和各级驻肇单位为肇庆发展作出积极贡献。

　　一年来，我们突出抓好管理服务创新，加快转变政府职能，政府自身建设扎实推进。

　　依法行政水平不断提升。向市人大及其常委会报告工作8次、提交各类专项工作报告23件，按期办理人大代表建议92件。办理政协提案130件。制定重大行政决策程序规定，实施重大行政处罚决定备案制度和行政执法案卷评查制度。获评全国法治城市创建活动先进单位。

　　行政管理改革不断深化。实施新一轮行政审批制度改革，取消、转移、下放、委托管理审批事项共233项，赋予高新区、新区第二批67项市级管理事权。成为全省企业登记相关领域审批制度改革试点。省网上办事大厅肇庆分厅开通运行。市行政服务中心审批业务办结时限比法定时限平均提速74%，公众满意度达99.8%。食品药品监管和卫生计生部门机构改革启动实施。市公共资源交易中心和公共资产管理中心挂牌运作。八成乡镇建立农村“三资”监管平台，“便民通”政务服务平台在全市乡镇推广，镇级社会综合服务平台实现全覆盖。

　　勤政廉政建设不断加强。严格落实中央八项规定，压减会议、检查和评比活动，清理规范机关办公用房，“三公”经费支出减少12.6%。深入开展“机关作风建设年”活动，深化政风行风评议，作风明查暗访常态化，着力整治“庸懒散奢”等不良风气。健全领导定期接访下访包案制度，市领导共接访928批次2631人次。政务公开走向制度化，创办《市民问政》电视节目和政务微博。行政监察和审计监督得到强化，政府投资项目廉政风险防控制度常态化实施，惩治和预防腐败体系更加健全。

　　回顾过去一年政府工作取得的新成效，我们深深感到，这是省委、省政府和市委正确领导的结果，是市人大、市政协监督支持的结果，是全市人民团结奋斗和社会各界凝心聚力的结果。我代表市人民政府，向全市人民，向各位人大代表、政协委员，向所有关心支持参与肇庆建设发展的外来投资者、港澳台同胞、海外侨胞和国际友人致以崇高的敬意和衷心的感谢！

　　同时，我们也清醒认识到，我市经济社会发展还存在一些困难问题，主要表现在：人均生产总值与全国同期平均水平仍有差距，统筹城乡区域协调发展力度有待加大；主导产业集聚度和竞争力还不高，生产性服务业发展滞后；城市化整体水平偏低，产城融合发展有待加快；空气质量有待提高，生态环境保护压力加大；基本公共服务与群众要求还有不少差距，改善民生任务依然艰巨；政府职能还需进一步转变，行政效能建设仍需加强。我们将高度重视，采取切实有效措施加以解决。

　　2014年工作安排

　　当前，经济形势仍然复杂多变，经济下行压力依然存在。但经济持续健康发展的基本面没有改变，特别是党的十八届三中全会作出了全面深化改革的战略部署，我省大力推进珠三角“九年大跨越”和粤东西北加快崛起，为我市破解发展难题、推动改革发展提供强大动力。只要我们解放思想，坚定信心，勇于改革，攻坚克难，就一定能够开创科学发展新局面。根据省委、省政府和市委的决策部署，今年政府工作的总体要求是：全面贯彻落实党的十八大、十八届三中全会、中央经济工作会议和省委十一届三次全会精神，按照市委十一届四次全会的部署，深入贯彻《珠三角规划纲要》，大力实施“两区引领两化”战略，坚持把发展作为第一要务，坚持把改革贯穿于经济社会发展各个领域，坚持把加快发展与提高效益、生态建设紧密结合起来，坚持把改革发展成果更多更公平惠及人民群众，统筹抓发展、促转型、优环境、惠民生、保稳定，确保人均生产总值赶超全国同期平均水平，为建设珠三角连接大西南枢纽门户城市打下更坚实基础。今年经济社会发展主要预期目标如下：地区生产总值增长12%，人均生产总值增长11%，规模以上工业增加值增长17.8%，地方公共财政预算收入增长15%，固定资产投资增长17%，社会消费品零售总额增长12%，实际吸收外资增长5%，外贸进出口总额增长6%，城镇居民人均可支配收入增长9%，农村居民人均纯收入增长10%，居民消费价格涨幅控制在3.5%以内，人口出生率和自然增长率控制在省下达计划以内，城镇登记失业率控制在2.6%以内，节能减排约束性指标完成省下达计划。重点抓好以下工作：

　　一、以实现赶超为标杆，着力增强经济综合实力。充分发挥投资的关键作用、出口的支撑作用和消费的拉动作用。全年安排重点项目100项，计划投资361.52亿元，其中计划投产10项、续建50项、新开工40项。继续抓好交通基础设施建设大会战，确保贵广和南广铁路肇庆段、肇花高速、二广高速怀集至连州段建成通车，加快推进广佛肇高速、广佛肇城际轨道、火车站综合体、客运总站、肇庆东站等综合性交通枢纽工程，力促汕昆和汕湛高速肇庆段年内动工，抓紧开展柳肇铁路和怀阳高速肇庆段前期工作。全年安排现代产业项目59项，计划投资135.96亿元，加快推进风华高端电子、三浦重工、维信橡塑等新建扩建重点项目，推动国电热电联产项目达标达产，协调加快大唐国际、中电国际热电联产项目和一批风电项目建设。落实加工贸易转型升级活动计划，发展壮大服务外包产业和外贸综合服务企业，打造外贸转型升级示范基地，加快建设电子口岸，创新实施“三个一”通关模式，提高优势出口产品档次和附加值，争取建设进口商品交易中心，力促外贸进出口稳定增长。积极培育行业性电子商务平台，推动本地优势产品“网上行”。推进万村千乡市场升级改造和农超对接，抓好城东新区等新城区商业网点规划建设，加快推进城东新区城市综合体、广佛肇城际轨道鼎湖站综合开发等重点商贸项目。加快建设旅游综合改革示范市，推动星湖风景名胜区创建5A景区，推进七星岩北门广场、鼎湖山新入口广场等项目建设。优化房地产业结构，促进房地产业持续平稳发展。

　　二、以深化改革为动力，着力激发体制机制活力。抓紧落实省13项年度重点改革任务，切实抓好市15项重点改革工作。全面完成商事登记制度改革，探索实施“非禁即入”的企业投资项目网上在线备案制，支持社会资本进入特许公共经营领域，在肇庆高新区和肇庆新区试点推行投资负面清单制度。建立全口径的预算编报体系，推行财政预决算信息公开制度，探索实施财政股权式投资。稳步推进“营改增”试点。支持优质企业通过改制上市、新三板挂牌等方式拓宽融资渠道。大力引进各类金融机构设立分支机构，支持发展普惠金融。创新城市建设投融资方式，大力引进保险、信托等长期资金和战略合作伙伴投资城市建设，建立有效防控债务风险的融资平台，争取发行市政债券。协同加快珠江—西江经济带一体发展，积极参与编制实施珠三角新的五个一体化规划，提升广佛肇、肇梧、肇贺战略合作水平，深化肇港澳台合作，积极拓展与东盟的合作。探索建立城乡统一的建设用地市场和农村产权流转交易市场，开展农村土地承包经营权确权登记颁证试点，稳步推进“土地股份化”改革。深化农村综合改革，力争山区县全部乡镇基本建立“三资”管理交易平台、60%的行政村建立社会综合服务平台。开展国有林场分类改革，引导各类市场主体参与国有林场生态公益林建设。

　　三、以集聚创新为路径，着力推动新型工业化。强化落实“一核、两带、三板块”工业总体布局和“4+3+2”工业主导产业培育发展措施，组织实施产业链延伸配套工程。加快肇庆高新区转型发展，深化“一区多园”合作，实现大旺产业转移园“二次扩园”，启动中心服务区规划建设，同步推进重大项目引进和现有产业改造升级。坚持“企业进园区、园区近城镇”发展，加快各地特色产业园区扩能增效，力争更多县（市）区建成省级产业园、每个园区形成若干特色主导产业。着力实施高成长性企业“倍增工程”，发展壮大50家可在三年内实现主营业务收入翻番企业，培育一批年主营业务超10亿元、30亿元、50亿元企业，力争实现超百亿企业“零”的突破。深入开展“送政策下基层进企业”活动，组织实施中小企业信息化推进工程和民营企业素质提升工程，支持中小微企业发行融资集合债券，促进中小微企业扩产增效。完善产学研用协同创新体系，着力打造主导产业公共服务平台，加快建设长春理工大学国家大学科技园肇庆分园和肇庆学院大学科技园，推进产业集群联合创新。积极打造智慧肇庆，推进光纤入户、WiFi热点覆盖和4G网络建设。以推动技术标准化为重点，积极创建省质量强市示范市。推动肇庆新港、三榕港打造成为西江物流枢纽港，扶持发展物联网应用、文化创意等新兴产业，加快“双龙”产业城、华南智慧城、中巴软件园和云服务产业园建设。严格项目能耗准入管理，加快节能改造。

　　四、以扩容提质为方向，着力推进新型城市化。全面贯彻落实中央城镇化工作会议精神，组织开展新型城镇化体制机制改革试点。优化城市布局和形态，修编完善肇庆市城市总体规划和历史文化名城保护规划，统筹推进“一江两岸”整体城市设计，抓好重点片区控制性详细规划和管网管线规划。深化肇庆新区城乡一体化综合配套改革，推动中央绿轴生态城开发建设，重点抓好政文组团市政道路等项目。大力推进中心城区基础设施建设，加快实施阅江大桥、鼎湖大道、大旺至端州一级公路改造、下湾城市公园等城际衔接项目，规划建设崇禧塔、阅江楼、梅庵三大历史文化街区，推进江滨西堤示范段等“三旧”改造项目，开展城中村改造整治试点，统筹开发城市地上地下空间。探索构建县域新城和中心镇产城融合发展机制，加快县域新城区扩容建设，推动怀集建设肇庆城市副中心，编制实施特色名镇总体发展规划和100条行政村规划，对特大镇赋予县级经济管理权限，增强中心镇衔接城乡功能，促进小城镇专业化、集约化和特色化发展。优化城际公共交通规划，增加跨市公交线路。继续推进城市管理重心下移，提高城市管理精细化、智能化水平。分类放开城镇落户政策，稳步推进城镇基本公共服务常住人口全覆盖，促进农业转移人口市民化。

　　五、以重大平台为载体，着力提高招商引资水平。强化落实招商引资责任制，建立招商项目统筹分享和协调跟踪机制，推动省市协同招商、园区联合招商和主导产业招商，创新实施小分队专业招商方式，重点引进强链补链大项目。突出抓好肇庆高新区招大引强聚智，重点引进世界和中国500强的总部项目、龙头项目。大力推进肇庆新区战略产业招商，重点引进节能环保、健康养生、文化创意等产业重大项目。推动粤桂合作特别试验区纳入《珠江—西江经济带发展规划》，创新建设管理开发机制，加快基础设施建设，扩大引进优质项目。加快广佛肇（怀集）经济合作区整合发展，积极开展联合招商，促进主导产业集聚发展。

　　六、以惠农增收为重点，着力加快农业现代化。全面贯彻今年中央1号文件精神，促进农业增效、农民增收。开展粮食稳定增产行动，建设44万亩高标准基本农田和14个国家级万亩粮食高产示范区。推进以种业为重点的农业科技创新，组建基层农产品质量检测站，抓好“菜篮子”基地和标准化规模养殖场建设，推广畜牧业生态健康养殖，扶持家禽业稳定发展，提高柑桔、花卉苗木、南药等特色种植业规模化、标准化水平。积极发展农业合作经济组织，培育10家以上农业龙头企业、创建20家市级农业股份合作示范社。发展壮大珍贵树种、良种油茶、竹子等特色林产业和林下经济。推进景丰联围加固三四期、56宗病险水库除险加固和中小河流治理等水利工程。继续实施农村电网改造升级和公路硬底化建设，提高农村公共服务水平。强化产业扶贫，构建脱贫致富长效机制。

　　七、以生态环保为抓手，着力建设宜居美丽家园。编制实施国家生态市创建规划，严格按照主体功能定位划定生态保护红线。落实“十二五”后半期主要污染物总量减排行动计划，实行区域及排污单位污染物排放总量控制制度。积极探索环境治理市场化机制，稳步实施环保第三方监管和治理。加大空气质量综合治理力度，推动高耗能行业燃煤企业改用天然气，抓好陶瓷等行业整治改造，强化“黄标车”清理淘汰、城市扬尘防治和工业锅炉整治。实施最严格水资源管理，完成星湖水质整治首期工程，加快新区污水处理厂、高新区污水处理二厂等项目建设并完善配套管网，城镇污水处理率力争提高到83%。严厉打击违法排污行为，坚决依法关闭不符合环保排放要求的重污染企业。完善环境应急体系和污染防治区域联动机制，增强环境监察监测能力。编制实施市域环卫专项规划，加快市垃圾发电厂、市生活垃圾无害化处理场和市医疗废物处理站新站建设，新创省卫生镇6个，新增省卫生村受益人口10万人以上。加快实施“森林之都·美丽肇庆”建设规划，开展新一轮绿化肇庆大行动，新增森林面积8.94万亩、森林蓄积258.27万立方米，新造生态景观林带11.44万亩、森林碳汇8.4万亩，推进羚羊山古栈道文化森林公园、肇庆新区中央绿轴道路绿化、高要紫云路森林景观大道建设，实施103条村庄绿化美化，扎实做好国家园林城市复查迎检，力争创建成为国家森林城市。加强用地准入管理，坚决遏制违法违规用地，积极盘活闲置低效用地，大力实施城乡建设用地增减“双挂钩”。强化地质灾害治理。

　　八、以民生福祉为根本，着力发展社会各项事业。创新城乡公共就业创业服务体系，促进以高校毕业生为重点的青年就业创业和农村转移劳动力、城镇困难人员、退役军人就业。抓好社保续保扩面，制订实施城乡居民参保激励政策，完善养老保险待遇正常调整机制，探索构建社保关系转移接续机制，健全重特大疾病医疗保险和救助制度。提高城乡最低生活保障标准，把年人均收入低于3600元的农村居民和低于5640元的城镇居民及城镇“三无人员”全部纳入低保救助范围。加大敬老院建设力度，发展老年服务产业，完善社会养老服务体系。健全保障性住房建设和分配机制。实施创建教育现代化先进市六大工程，实现公办义务教育规范化学校全覆盖，探索实施公办学校校长教师交流轮岗，完善义务教育学校免试就近入学制度，保障异地务工人员随迁子女接受义务教育。继续支持肇庆学院“申硕改大”，推动肇庆医专、科技学院和工商学院升格本科院校，积极引进省部高等院校进驻办学，鼓励社会力量兴办教育。深化医药卫生体制改革，抓好德庆、封开第二批县级公立医院综合改革试点。推进基层医疗卫生机构综合改革，加快肇庆新区医院建设和县级医院改扩建，启动乡镇卫生院500套医务人员公租房建设。加强重大疫情监测和防控。鼓励社会资本发展健康服务产业。提高出生人口素质，落实“单独二孩”生育政策。深化文化体制改革，加快推进包公文化园、端砚文化产业园等项目，抓好乡镇综合文化站建设，支持具有本地特色的文化精品创作，鼓励创办非公有制文化企业。推动中华优秀传统文化教育进社区、进校园、进课堂。深入开展“美丽肇庆·幸福家园”文明创建活动，力争达到全国文明城市各项创建标准。启动第十五届省运会筹备工作，扎实备战第十四届省运会，规划建设社区体育公园。全面推行社区网格化管理和社会化服务，实行网上受理信访制度，健全及时就地解决群众合理诉求机制，依法有效预防化解社会矛盾。建立最严格覆盖全过程的食品药品监管制度，实行食品原产地可追溯制度和质量标识制度。健全落实安全隐患排查治理和预防控制机制，坚决遏制安全事故发生。继续推进“平安肇庆”建设，健全立体化治安防控体系，严厉打击违法犯罪活动。健全应急管理机制。加强国防动员和人防工作，争创全国“双拥”模范城。继续做好第三次全国经济普查。充分发挥民族、宗教、外事、侨务、港澳、地方志、档案、气象、水文、地震、慈善、妇女儿童、残疾人等工作的重要作用。

　　九、以转变职能为关键，着力提升法治服务水平。自觉接受人大及其常委会法律监督和政协民主监督，定期向人大及其常委会报告工作和向政协通报情况，建立重大决策出台前向人大报告制度，高质量办好人大代表建议和政协委员提案。认真听取各民主党派、工商联、无党派人士、人民团体、专家顾问的意见建议。坚持问政于民、问需于民、问计于民，严格落实重大决策风险评估和公众参与制度。继续推进市县政府职能转变和机构改革，强化政府公共服务、市场监管、社会管理、环境保护职责，深入推进事业单位分类改革，积极发展社会组织，扩大政府购买社会服务。深入实施城市综合执法和统一执法。完善行政复议机制，落实行政应诉规则。制定公开政府职能部门权责清单，抓紧制订行政审批保留事项目录并实行动态管理，继续依法向基层下放事权，实现县级以上政府行政审批事项比2011年底减少40%以上。加强省网上办事大厅肇庆分厅与各县（市、区）以及各部门的对接运行，上半年建成乡镇（街道）网上办事站。健全社会普法机制，推进法律服务进村进社区。创新调查研究、政务公开和网络问政等联系群众服务群众制度，办好12345政府服务热线。扎实开展党的群众路线教育实践活动，全面落实中央“八项规定”精神和《党政机关厉行节约反对浪费条例》，深入开展行风评议，强化明查暗访常态化，切实解决“四风”突出问题，提高公务员执行力。完善惩治和预防腐败体系，加强行政监察和审计监督。推进公务用车制度改革，严控“三公”经费支出。

　　各位代表，在落实省十件民生实事有关任务的同时，市政府将全力办好今年十件惠民实事：一是提升就业社保水平。新增城镇就业46600人，失业人员再就业12000人，促进创业2650人，组织劳动力转移减免费培训32000人。完成全市市民社会保障卡发放，基本实现社会保障卡户籍人口全覆盖，并以此为载体开通政府“一卡通”公共服务。二是实现教育强镇全覆盖。创建省教育强镇8个（广宁县2个、封开县2个、怀集县4个）。三是提高底线民生保障。适时启动低收入群众临时价格补贴与价格上涨联动机制，保障低收入群体和困难群众临时价格补贴及时足额发放。建设30个农村幸福院示范点。落实低保残疾人生活津贴和重度残疾人护理补贴制度。四是加强医疗卫生服务。市级财政统筹2000万元，用两年时间实现全市乡镇卫生院设备装备“五个一”目标。90%以上乡镇卫生院能提供中医药服务。参加免费孕前优生健康检查目标人群覆盖率达80%以上。五是推进保障住房建设。新增保障性住房4649套（户），基本建成保障性住房和棚户区改造住房共4507套（户）。继续推进水上居民安居工程，年内计划安置514户。六是实施平价惠民工程。扶持建立一批农超对接示范点、平价商店和农民合作组织农（副）产品直销店，构建与20个小区、40个农民专业合作社和种养基地对接的农（副）产品配送服务平台。七是优化公共文体服务。动工建设市博物馆新馆、市文化馆新馆、市档案馆、市少年宫，实施全民阅读工程。推进公共体育服务均等化，建设10个乡镇农民体育健身工程。启动第十五届省运会新体育场馆规划建设。八是推进城乡道路改造。加快推进端州城区“断头路”建设，启动紫云路、新园路（信安路—端州一路）、黄岗路（砚都大道以北—紫云路）、黄岗四路西段、建设二路共5条道路建设。完成全市农村公路硬底化建设300公里。九是加强生态工程建设。完成第三批40条名村、35条示范村创建工作，鼎湖区、四会市、高要市、广宁县、德庆县、封开县、怀集县创建市级以上生态村（镇）各不少于2条（个）。完成市垃圾填埋场扩容（二期）工程建设，建成农村生活垃圾收运处理体系。建设北岭山森林步梯公园，规划面积510公顷，建设3条登山步道、1个主入口广场及配套设施。十是加强防灾减灾建设。全面完成市三防指挥部决策系统升级改造，在肇庆城区建设5个安全气象社区试点，每个社区设立一个气象灾害防御服务站。

　　各位代表，谱写伟大“中国梦”的肇庆篇章需要全市人民的共同努力。我们要紧密团结在以习近平同志为总书记的党中央周围，按照省委、省政府的决策部署，在中共肇庆市委的坚强领导下，齐心协力，锐意改革，务实创新，开拓进取，为加快建设成为珠三角连接大西南枢纽门户城市而努力奋斗!
